# PhyInformR: phylogenetic experimental design and phylogenomic data exploration in R

**DOI:** 10.1186/s12862-016-0837-3

**Published:** 2016-12-01

**Authors:** Alex Dornburg, J. Nick Fisk, Jules Tamagnan, Jeffrey P. Townsend

**Affiliations:** 1 North Carolina Museum of Natural Sciences, Raleigh, North Carolina 27601 USA; 2 Department of Biostatistics, Yale University, New Haven, Connecticut 06510 USA; 3 Center for Infectious Disease Modeling and Analysis, Yale School of Public Health, Yale University, New Haven, Connecticut 06510 USA; 4 Department of Ecology and Evolutionary Biology, Yale University, New Haven, Connecticut 06525 USA; 5 Program in Computational Biology and Bioinformatics, Yale University, New Haven, Connecticut 06511 USA

## Abstract

**Background:**

Analyses of phylogenetic informativeness represent an important step in screening potential or existing datasets for their proclivity toward convergent or parallel evolution of molecular sites. However, while new theory has been developed from which to predict the utility of sequence data, adoption of these advances have been stymied by a lack of software enabling application of advances in theory, especially for large next-generation sequence data sets. Moreover, there are no theoretical barriers to application of the phylogenetic informativeness or the calculation of quartet internode resolution probabilities in a Bayesian setting that more robustly accounts for uncertainty, yet there is no software with which a computationally intensive Bayesian approach to experimental design could be implemented.

**Results:**

We introduce PhyInformR, an open source software package that performs rapid calculation of phylogenetic information content using the latest advances in phylogenetic informativeness based theory. These advances include modifications that incorporate uneven branch lengths and any model of nucleotide substitution to provide assessments of the phylogenetic utility of any given dataset or dataset partition. PhyInformR provides new tools for data visualization and routines optimized for rapid statistical calculations, including approaches making use of Bayesian posterior distributions and parallel processing. By implementing the computation on user hardware, PhyInformR increases the potential power users can apply toward screening datasets for phylogenetic/genomic information content by orders of magnitude.

**Conclusions:**

PhyInformR provides a means to implement diverse substitution models and specify uneven branch lengths for phylogenetic informativeness or calculations providing quartet based probabilities of resolution, produce novel visualizations, and facilitate analyses of next-generation sequence datasets while incorporating phylogenetic uncertainty through the use parallel processing. As an open source program, PhyInformR is fully customizable and expandable, thereby allowing for advanced methodologies to be readily integrated into local bioinformatics pipelines.

Software is available through CRAN and a package containing the software, a detailed manual, and additional sample data is also provided freely through github: https://github.com/carolinafishes/PhyInformR.

**Electronic supplementary material:**

The online version of this article (doi:10.1186/s12862-016-0837-3) contains supplementary material, which is available to authorized users.

## Background

The 21^st^ century has witnessed rapid progress in the phylogenetic resolution of the evolutionary relationships in the Tree of Life, associated with a trend toward analysis of multi-locus and even genome-scale datasets [1–4]. However, despite this wealth of data, achieving resolution of some key nodes in the tree of life remains challenging. Some nodes persistently elude any resolution; others are characterized by conflicting results, each based on different sets of data, and each well-supported by current metrics of support [5, 6]. A critical step towards resolving and stabilizing our understanding of the Tree of Life is the development of tools that identify potential sources of non-phylogenetic signal—parallelism or convergence in character state that do not reflect shared evolutionary history—on phylogenomic datasets [5, 7].

Analyses of phylogenetic informativeness (PI; [8]) and quantification of quartet internode resolution probabilities (QIRP) [9, 10] provide a framework with which to predict the phylogenetic utility of large multi-locus and phylogenomic datasets. These analyses provide insights into the predictive utility of sequences across entire topologies as well as for individual nodes. For example, the shape of PI profiles can be used to determine either the potential utility of data for inference or the severity to which phylogenetic information content in a dataset has decayed over the temporal history of a clade [8, 11]. Although a useful heuristic, PI profiles are based solely on rates of character change and therefore provide no direct prediction of how homoplasious site patterns will influence phylogenetic resolution of specific nodes [11, 12]. However, QIRP calculations make use of theory that posits a predictive relationship between the rate of evolution, specified internode distances, and tree depth with the probability of resolving a given node thereby allowing for a more detailed prediction of how homoplasy will impact inference [9].

Using a rate of sequence evolution for an empirical dataset in conjunction with an *s*-state Poisson model (*s* ≥ 2), Townsend et al. [9] derived a model that calculates the probability of resolution for a given phylogenetic quartet. These calculations quantify not only the probability of correct resolution (QIRP), but also quartet internode homoplasy probabilities (QIHP) that represent the probability of having greater strength of support at a given internode for an incorrect rather than correct quartet topology as well as quartet internode polytomy probabilities (QIPP) that represent the probability of no resolution [9]. These calculations can empower sequencing pipelines to generate data that will resolve specific problems. Specifying a range of possible tree depths and internodes with estimated rates of markers, investigators can rank the effectiveness of candidate loci, saving both time and sequencing costs. Although the development of these methods targeted phylogenetic experimental design prior to sequencing [8], they have been successfully applied as data filtration metrics [13] and used to assess the validity of inferences based on existing markers [3, 14]. This additional utility makes these calculations potentially very useful in bioinformatic pipelines aimed at selecting loci from existing genomic datasets for analyses. However, software enabling the application of these tools is extremely limited.

The only tools currently available for analyses of phylogenetic informativeness are the locally implementable program TAPIR [15], which only generates PI profiles, and the PhyDesign web application [16], which has a modest server-based computational throughput and a limited scope of functionality. While these applications are useful, their limitations are stymieing adoption of phylogenetic informativeness and quartet probability based methods. There are powerful theoretical advances not reflected in these applications. For example, quantification of QIRP or QIHP in existing software assumes an underlying Jukes-Cantor nucleotide substitution matrix, while theory has been derived that facilitates a more accurate calculation that can be conducted with any substitution model [17]. Furthermore, existing applications force an assumption of equal divergence times for all four taxa in a phylogenetic quartet. This assumption is commonly not met in empirical datasets [18], and theory has been derived that facilitates calculations for any set of branch lengths [19]. A third major limitation of current implementations is that there is no way to integrate over branch length uncertainty using Bayesian posterior distributions. Finally, the ability to visualize results with previous tools is modest and does not meet the needs of investigators with phylogenomic or next-generation sequence data.

Publicly available software that addresses these three criteria is a critically needed resource for assessing the utility of potential or actual data sets for phylogenetic inference. However, open source software facilitating both advanced calculations and visualizations of information content has been lacking. Here we present the software package PhyInformR, an open source program—designed to keep pace with advances in theory—that will allow users to quantify and display the phylogenetic information of computationally demanding datasets.

## Implementation

PhyInformR is an open source software package in R [20] that utilizes the phylogenetic packages APE [21], PhyTools [22], and Geiger [23]. As the expanded theory enabling any substitution model and uneven quartet trees [17, 19] require solving symbolic equations. To facilitate faster throughput, parallel processing is supported using the foreach software package [24]. PhyInformR can be downloaded along with an in depth user guide and example phylogenies [25–27] from github: https://github.com/carolinafishes/PhyInformR. As an input, PhyInformR uses site rates estimated from software such as Hyphy [28] and user tree topologies to rapidly enable quantification of PI across datasets or user defined dataset partitions.

Rather than being a black box, the flexibility of the R language allows users to rapidly define any subsets of their data to quantify all metrics available in the phyDesign web interface [16] such as PI profiles [8] or QIRP, QIPP, or QIHP values [9]. PhyInformR expands the power of the phyDesign web interface [16] by incorporating recently published theory that calculates metrics on user specified quartets with uneven branch lengths [19] using any specified symmetrical substitution model and any empirical distribution of base frequencies [17]. The software also accommodates phylogenetic uncertainty, enabling these calculations to be performed across Bayesian posterior distributions of user supplied tree topologies and branch lengths. To overcome challenges in the visualization of information content in phylogenomic datasets, PhyInformR additionally offers a flexible suite of options (Additional file 1) for users to view quantified values of QIRP, QIPP, or QIHP that utilize customizable graphics packages such as ggplot2 [29].

## Results and discussion

### Expanding computational throughput

**Fig. 1:**
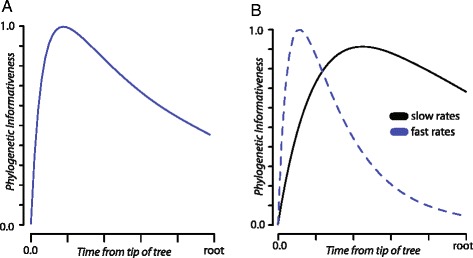
Phylogenetic informativeness profiles generated in PhyInformR based on a selection of 60 loci from [3] for (**a**) all loci; (**b**) a user defined partition of “fast” versus “slow” rates of nucleotide substitution, with the upper 10% of the rate distribution selected as “fast”

We used the avian tree topology from Prum et al. [3] and corresponding site rates estimated using Hyphy [28] to quantify and plot PI profiles [16] (Fig. 1a). PI profiles of the concatenated data demonstrate a slow decline towards the root of the tree, indicative of increased homoplasy [11, 13]. By defining two partitions based on two categories of site-rates, visualizations of PI profiles demonstrate the limited utility of faster evolving sites for deeper nodes (Fig. 1b). Explorations of this type, such as additional evaluations of individual loci or automatically generated partitions of site rate vectors by codon position (provided the generating alignment was in frame) allow for both dynamic adjustments of plots and assessing the impact of site removal on PI profiles. This type of interactive data exploration was not previously possible and is of particular utility for estimation of divergence times. Finding dataset partitions that minimize decay in PI profile has been suggested to aid in mitigating the impacts of branch length estimation errors in divergence dating analyses [13]. Given the computational expense of estimating divergence times using phylogenomic scale data, identifying loci with the lowest declines of PI offers a potential data selection criteria for divergence time analysis pipelines [3].

**Fig. 2:**
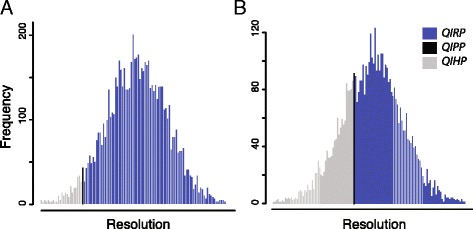
Graphical output of the Monte Carlo based analysis of QIRP (*blue*), QIPP (*black*), and QIHP (*grey*) shown from right to left respectively. Quantification was based on a selection of 60 loci from [3] for the most recent common ancestor of *Opistocomus hoatzin* and other birds. **a** Results from the slower site rate partition depicting a higher probability of correctly resolving this node. **b** Results from a fast site partition demonstrating a wider spread of potential support for the correct or incorrect topology, depicting the elevated risk of spurious results from this partition

Although PI profiles provide a useful visualization, it is important to note that PI profiles perform no explicit quantification of how homoplasy will impact tree topology [11]. Prediction of the impact of homoplasy can be conducted by calculation of QIRP and QIHP [8, 9, 17]. Due to the computational demands of the Monte Carlo approach to solving for the probabilities presented in Townsend et al. [9], the phyDesign web interface enables download links only for user-defined nodes of interest, and only computes results using a closed-form analytical approximation. This approach does not allow for interactive exploration of data partitions, and also does not yield the more nuanced graphical output of the higher moments of the resolution probability distribution [16]. In contrast, PhyInformR performs both the rapid analytical approximation and the intuitive Monte Carlo visualization (encoded to rapidly execute in parallel [24], if desired). For example, using the two partitions of Prum et al. [3] data examined above, we can compare the predicted probability distribution of the slower versus faster site rates for the most recent common ancestor of *Opistocomus hoatzin* and other birds. In this case, the slower site rate partition provides a higher probability of correctly resolving this node (Fig. 2a). Additionally, the fast site partition demonstrates a wider spread of potential support for the correct or incorrect topology, conveying both the potential for high support for a correct result and the elevated risk of spurious results when using this partition in phylogenetic analyses (Fig. 2b).

### Advanced calculations, Bayesian integration, and result visualization

Early implementations [9] assume a Jukes-Cantor model of substitution [30] as well as equal branch lengths in the quartet tree. Estimated model fits and tree topologies from empirical datasets suggest that these assumptions are commonly violated [18, 31–33]. PhyInformR encodes new theory allowing users to specify any symmetrical substitution model, and to specify uneven branch lengths in the quartet. Furthermore, PhyinformR can incorporate uncertainty in tree topology and branch lengths into these calculations. This integration over uncertainty of the underlying phylogenetic tree provides a major advantage in comparison to available software that only permits users to evaluate single tree topologies with fixed branch lengths [16]. Use of a single tree can be problematic since quantifications of QIRP, QIHP, and other PI based metrics are sensitive to topology and branch lengths [3]. PhyInformR enables users to iterate calculations over any set of hypothetical trees or over posterior distributions from Bayesian phylogenetic analyses. These parallelizable calculations enhance the robustness of QIRP, QIPP, and QIHP.

**Fig. 3:**
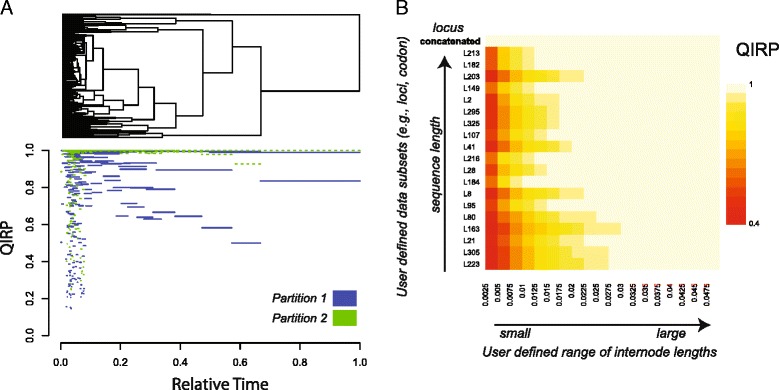
**a** Visualizations of QIRP as in [20], comparing QIRP calculations of two dataset partitions from [3] across the tree. Partition one represents “slower” evolving sites based on a cutoff of 0.003 subs/site per million years while partition two represents “faster” evolving sites that lie above the user defined cut off value. **b** QIRP heat map from [3] with probabilities of QIRP quantified across a range internode lengths for a shallow node. Each row corresponds with a user defined delimitation of site-rates (loci and a concatenated dataset in this example, though heatmaps can be modified to any user defined partitions). Shadings correspond with QIRP values. Arrows in the x and y axes respectively indicate internode or sequence length from small to large

**Fig. 4:**
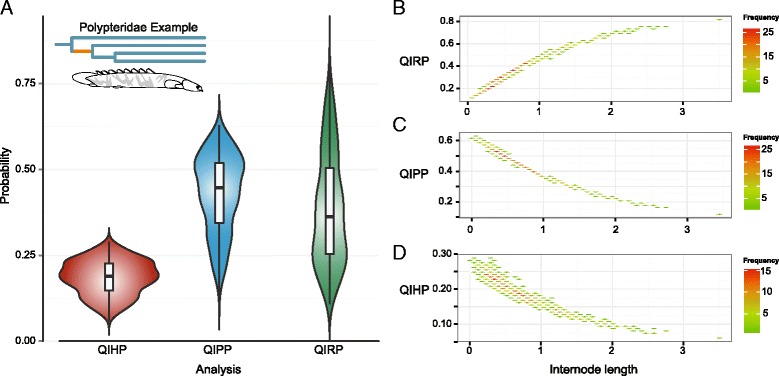
Two visualizations of Quartet internode resolution (QIRP), polytomy (QIPP), and homoplasy (QIHP) probability calculations in PhyInformR using a focal node of bichirs (most recent common ancestor of *Polypterus congicus* and *P. ansorgii)* with Bayesian time trees and RAG1 sequence data from Near et al. [18]. **a** Violin plots displaying the kernel probability density of each of the three calculations across the posterior distribution of trees for the focal node with box plots of the quartiles for each distribution overlaid within. **b**–**d** Frequency plots for comparing QIRP (**b**), QIPP (**c**), and QIHP (**d**) against the length of internode. All points are sampled from the posterior. Color indicates the frequency of the joint QI measure and internode length within the posterior. In the Near et al. [18] tree from which this internode came, this internode was unresolved; this plot demonstrates that in most samples from the posterior, internode length was short, conferring little power to resolve branching order

The ability to store these calculations into objects creates a foundation for numerous advances in visualization. For example, visualizing QIRP values across an entire tree for multiple dataset partitions simultaneously [34] allows for the assessment of whether a concatenated dataset with an overall low QIRP may contain several partitions with high QIRP that could be assembled to resolve a specific node. In the case of the Prum et al. avian dataset [3], we can see that one partition of the dataset has higher predicted utility for resolving more of the deep internodes of the avian Tree of Life, whereas another has much lower probabilities (Fig. 3a). To account for uncertainty in internode lengths, heatmaps can be generated to provide a graphical display of information content across a range of internode lengths, portraying trends in utility. For example, heatmaps of individual loci in the avian dataset (Fig. 3b) reflect a trend of decreasing signal at small internodes. These sorts of trends shed light on sources of gene-tree conflict, illuminating whether lack of power (high QIPP) or any combination of convergence and parallelism (high QIHP) are driving discordance. Further, calculation of QIRP, QIPP, and QIHP across posterior distributions of trees enables visualizations that convey how the predicted power of data to resolve a node changes with the degree of uncertainty present for a focal phylogenetic quartet topology and internode lengths. For example, an analysis of the RAG1 gene from a study of Bichirs [27] depicts nearly equal kernel probability densities between QIRP and QIPP for a node that was—indeed—not strongly supported in the original study (Fig. 4a). A frequency plot of internode lengths for each measure demonstrates that QIRP decreases at small internodes, while both QIPP and QIHP increase (Fig. 4b). Not only does the probability of homoplasy increase with shorter internode lengths, but small differences in the branch length of increasingly small internodes exerts increasingly larger effects on the predicted utility of a dataset (Fig. 4c & d). In this example, these results nevertheless lead to the expectation that the attempt to confidently resolve this particular node is underpowered.

## Conclusions

PhyInformR provides a new toolset in the phylogenetic toolbox that characterize phylogenetic information in next-generation sequence datasets, enabling both new approaches to experimental design and dataset scrutiny. For targeted phylogenetic studies, PhyInformR will allow groups of loci to be screened for their phylogenetic utility prior to sequencing, potentially cutting costs and time. Likewise, screening loci during probe set development in next-generation sequencing bioinformatic pipelines can cut sequencing costs and the necessity for data filtration during downstream analyses. Furthermore, PhyInformR can complement investigations of topological or branch length incongruence, and can provide insight into sources of error, in some cases facilitating conclusive resolution of nodes that would otherwise remain contentious. The flexibility of graphical output in R makes PhyInformR an expansible tool set for dataset exploration allowing continued development of approaches to visualizing trends in genome-scale datasets. Such capabilities are critical not only to better our understanding of sources of topological incongruence, but also to the goal of continuing to increase our ability to resolve a robust and accurate Genomic Tree of Life.

## Availability and requirements

PhyInformR is implemented in R with the package available on CRAN and at: https://github.com/carolinafishes/PhyInformR

## Additional file

Additional file 1: PhyinformR user guide and tutorial. (PDF 7055 kb)

## Abbreviations

PI: Phylogenetic informativeness
QIHP: Quartet internode homoplasy probability
QIPP: Quartet internode polytomy probability
QIRP: Quartet internode resolution probability
