# Supplementary material for: PhyInformR: phylogenetic experimental design and phylogenomic data exploration in R
Source: BMC Evol Biol. 2016 Dec 1;16:262. doi: 10.1186/s12862-016-0837-3 (PMC5134231; doi:10.1186/s12862-016-0837-3)
Supplement: Additional file 1: — PhyinformR user guide and tutorial. (PDF 7055 kb) [file 12862_2016_837_MOESM1_ESM.pdf]

phyinformR

user guide and tutorial

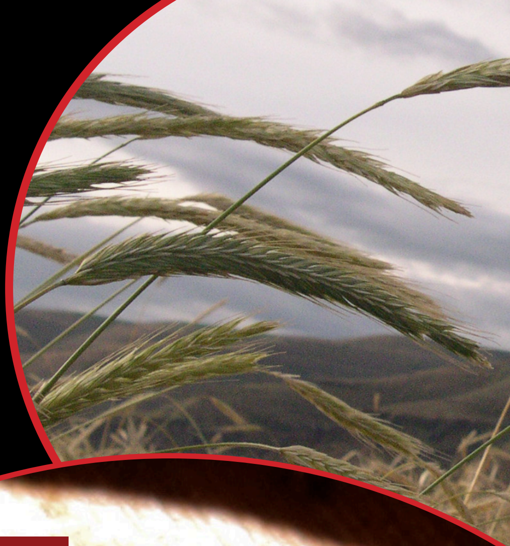

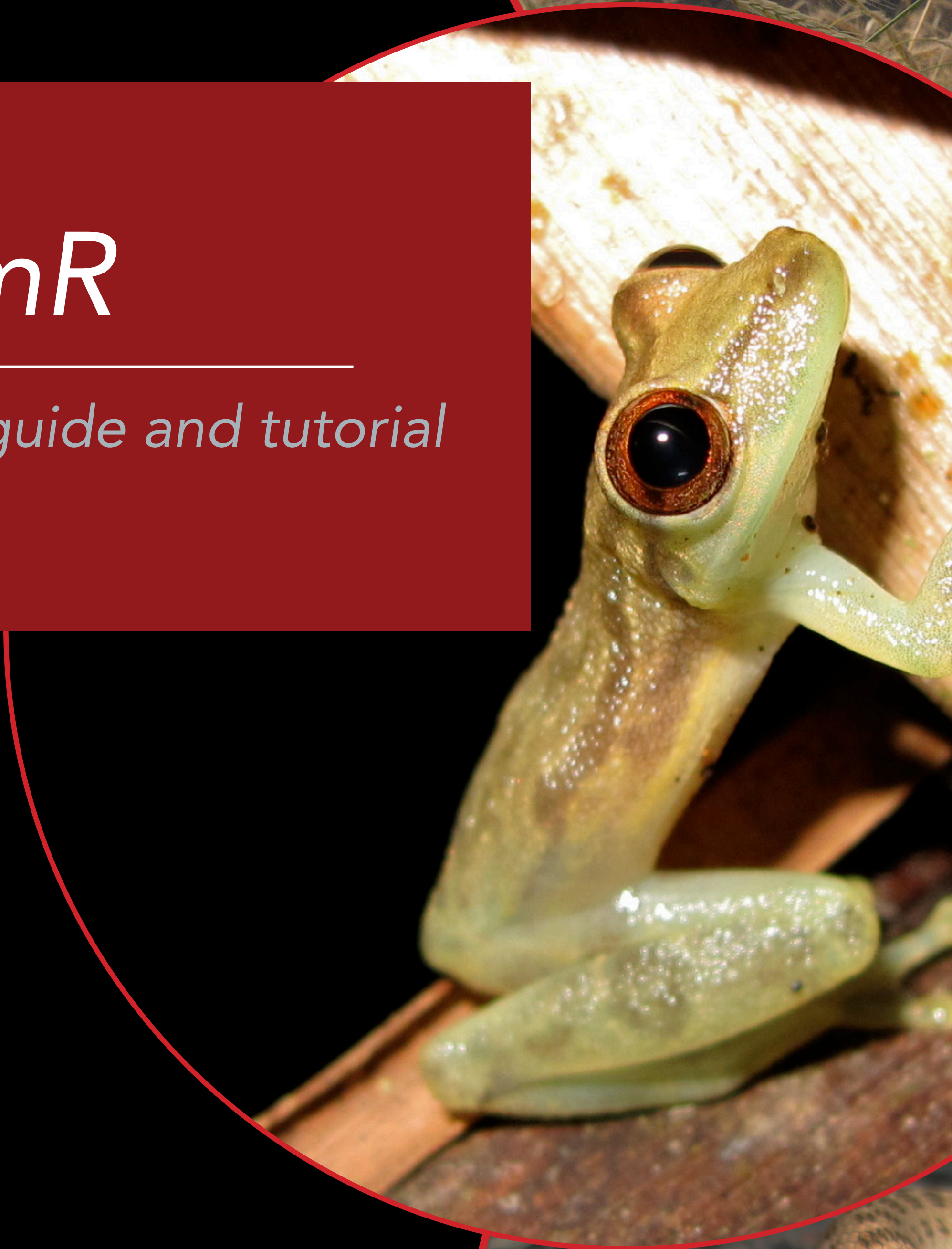

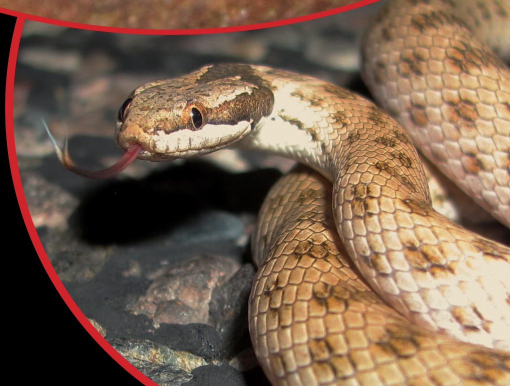

Dornburg • Fisk • Tamagnan • Townsend

Updates to phyinformR and the manual are available at
carolinafishes.github.io/software/phyinformR/

Please check your release version to make sure you are not missing out on new features

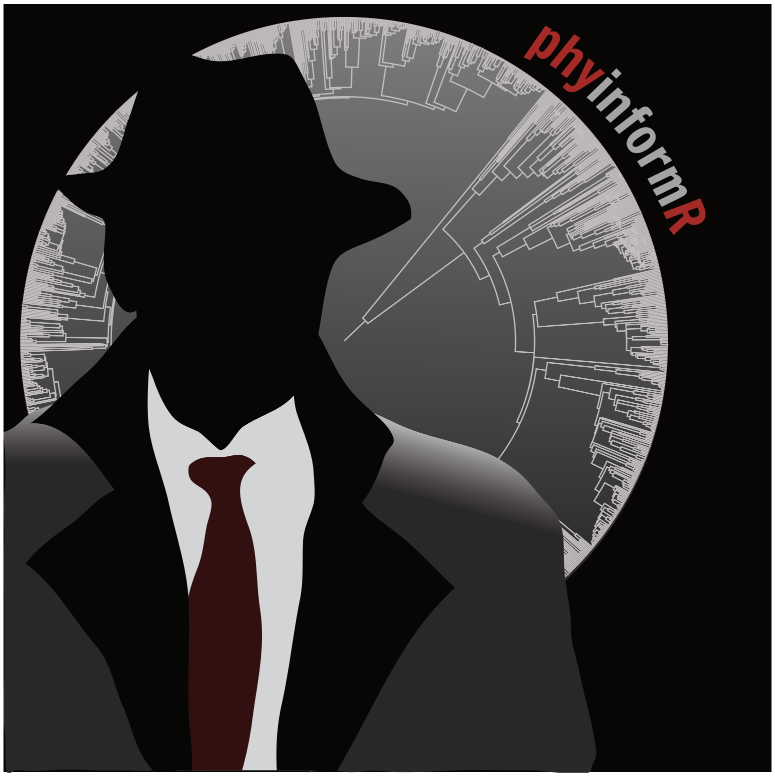

Copyright 2016 Alex Dornburg, J. Nick Fisk, Jules Tamagnan, and Jeffrey P. Townsend

This document is part of phyinformR

phyinformR is free software: you can redistribute it and/or modify it under the terms of the GNU General Public License as published by the Free Software Foundation, either version 3 of the License, or (at your option) any later version.

phyinformR is distributed in the hope that it will be useful, but WITHOUT ANY WARRANTY; without even the implied warranty of MERCHANTABILITY or FITNESS FOR A PARTICULAR PURPOSE. See the GNU General Public License for more details.

You should have received a copy of the GNU General Public License along with phyinformR. If not, see <http://www.gnu.org/licenses/>.

Images: Copyright 2016 Alex Dornburg

informR

Phylogenomic experimental design and data exploration in R

A. Dornburg, J. N. Fisk, J. Tamagnan, and J.P. Townsend

Table of Contents

Section 1
Installation … … … … … … … … …4
Dependencies … … … … … …4
Section 2
Phylogenetic informativeness profiles … … …6
Section 3
Resolution probability quantification … .11
Advanced resolution probability quantification … … .13
Posterior distributions … … … … .15
Section 4
Advanced visualizations … … … … … … .19
Section 5
Acknowledgements … … … … … … … … .25

Section

01

Installation initial setup and an overview of dependencies

Installation and setup

There are two easy ways to install PhyInformR

1) PhyInformR is hosted on CRAN

```
install.packages("PhyInformR")
library(PhyInformR)
```

2) PhyInformR is hosted on Github

```
library(devtools)
install_github("carolinafishes/PhyInformR")
library(PhyInformR)
```

Once you load PhyInformR, set the number of cores at the start of your session to enable later parallel processing:

```
library(doParallel)
registerDoParallel(cores=8)
```

Dependencies

PhyInformR would not be possible without the efforts of other developers. PhyinformR should automatically load all dependencies on start up (and install any that you do not already have installed) during the initial setup

Here is a brief list of the primary packages that supply the foundation for phyinformR:
ape[1], geiger[2], phytools[3], gplots[4], RcolorBrewer[5], foreach[7], iterators[7], doParallel[7], ggplot2[8]

NOTE FOR GITHUB INSTALL: devtools currently does not install all dependencies on certain versions of Windows. If errors are encountered during installation, manually install dependent packages as follows, then use devtools as above:

```
install.packages("doParallel")
install.packages("phytools")
install.packages("splines")
install.packages("gplots")
install.packages("RColorBrewer")
install.packages("foreach")
install.packages("iterators")
install.packages("geiger")
install.packages("doParallel")
install.packages("gridExtra")
install.packages("hexbin")
install.packages("PBSmodelling")
install.packages("ggplot2")
```

References:

1. Paradis E, Claude J, Strimmer K. APE: Analyses of Phylogenetics and Evolution in R language. Bioinformatics. 2004;20:289–90.

2. Harmon LJ, Weir JT, Brock CD, Glor RE, Challenger W. GEIGER: investigating evolutionary radiations. Bioinformatics. 2008;24:129–31.

3. Revell LJ. phytools: an R package for phylogenetic comparative biology (and other things). Methods Ecol. Evol. 2011;3:217–23

4. Warnes,G.R., Bolker, B., Bonebakker, L., Gentleman, R., Liaw, W.H.A., Lumley, T., Maechler, M., Magnusson, A., Moeller, S., Schwartz, B., Venables B., gplots: Various R Programming Tools for Plotting Data. 2016. R package version 3.0.1. https://CRAN.R-project.org/package=gplots.

5. Neuwirth,E.RColorBrewer: ColorBrewer Palettes. 2014. R package version 1.1-2. https://CRAN.R-project.org/package=RColorBrewer

7. McCallum E, Weston S. Parallel R. "O'Reilly Media, Inc."; 2011.

8. Wickham H. ggplot2: Elegant Graphics for Data Analysis. Springer; 2016.

Section

02

Profiling phylogenetic informativeness

phyinformR

Phylogenetic informativeness profiles

Townsend's[1] phylogenetic informativeness profiles are a visual tool that enables assessment of the predicted utility of a given sequence for phylogenetic inference across a timescale of interest

Use of this method requires two inputs:
site rates and a guide tree

Site rates can be obtained through a variety of software applications such as hyphy, rate4site, or DNArates. The phydesign web interface[2] makes quantifying site rates easy:

1) Navigate to http://phydesign.townsend.yale.edu/
2) Upload an alignment and ultrametric tree
3) Choose your program for estimating rates from a dropdown
4) Wait for the email that your results are ready

Once you have site rates, use the the "c" function in R to format them. You are ready to explore your data

Example:

```
mysiterates<-c(0.00034, 0.005678, 0.0,..., 0.008967)
```

GETTING STARTED

For this walkthrough, we will be using the avian tree and site rates from Prum et al.[3] that are distributed with PhyInformR

```
library(ape)
read.tree(system.file("extdata","Prumetal_timetree.phy",
package="PhyInformR"))->tree
```

The functions in phyinformR use sites rates as matrices, so first

```
as.matrix(prumetalrates)->rr
```

Then

```
informativeness.profile(rr,tree, codon="FALSE", values="off")
```

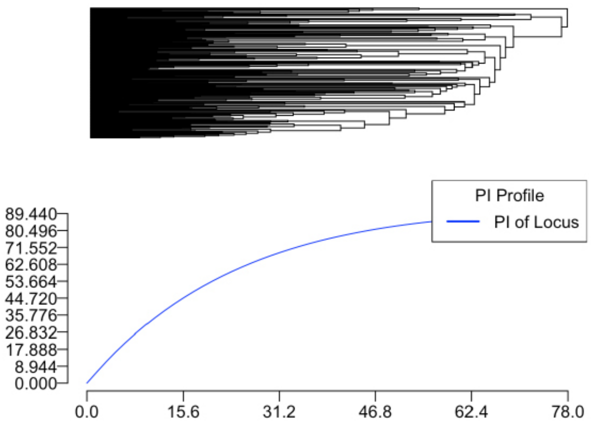

To obtain PI profiles for each codon position, you can toggle codon="TRUE" if you are in reading frame (note that this data is not!)

```
informativeness.profile(rr,tree, codon="TRUE", values="off")
```

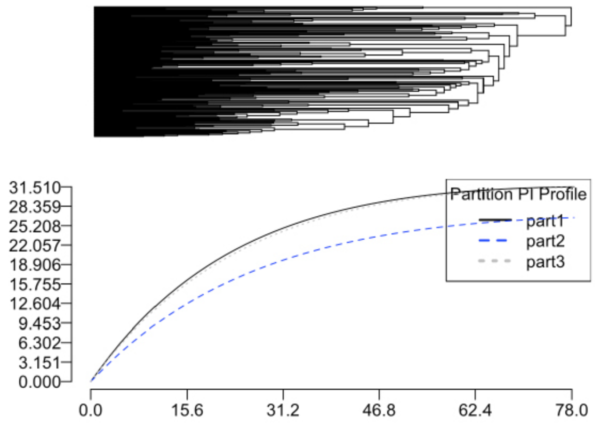

If you would like PhyInformR to output of branching times and PI values, simply switch the values="on"

Exploring Data with PI profiles

Let's do something different and partition the data by site rates. First we will view the rates:

```
hist(rr)
```

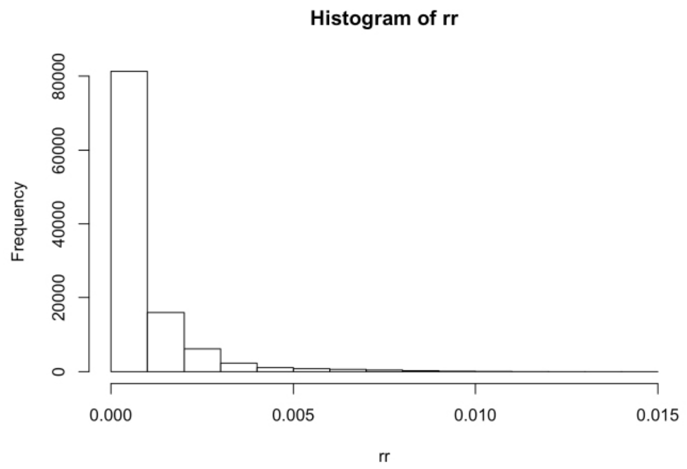

We can see a bit of a tail going out, lets see what happens when we partition the data by rates above and below (0.003). We'll start by creating some partitions

By defining rate based breaks in our data, we can see the PI of "fast" versus "slow" sites

Section 02

```
lower<-c(0,0.003)
upper<-c(0.0031,10)
cbind(lower,upper)->breaks
```

PhyInformR has a function allowing profiles to be broken along any point in the rate vector, to assess changes in phylogenetic informativeness associated with thresholding the dataset at that rate

```
multi.profile(rr,tree, breaks,values="off")
```

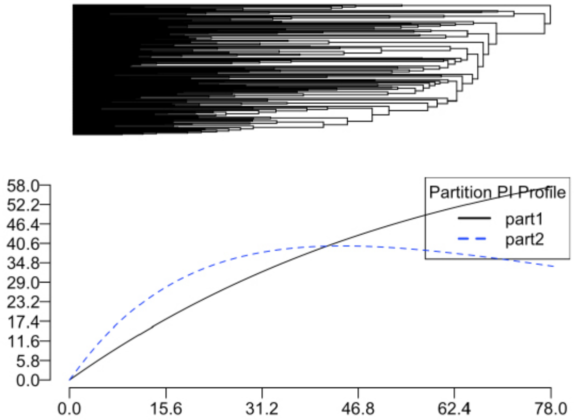

Partition 1 represents the slower site rates. As expected, the decay in phylogenetic informativeness for partition 1 is much lower across the tree than for partition 2. Conversely, we can see the faster sites in part two are informative for recent divergences, yet exhibit a rapid decline in informative site patterns as we move to deeper portions of the tree.

The above examples serve to illustrate what PhyInformR does, but this approach is not common practice. Instead, it is more common to work with character sets partitioned by loci you wish to evaluate. In this case, simply use the same approach as above to define your loci and use defined.multi.profile

In this example we will compare locus 1, that spans sites 1-1594 in the alignment and locus2, that spans sites 1595-2787.

```
Lower<-c(0,1594)
Upper<-c(1595,2787)
Breaks<-cbind(Lower,Upper)
```

Now we can visualize the profile using

```
defined.multi.profile(rr,tree,Breaks,values="off")
```

Section 02

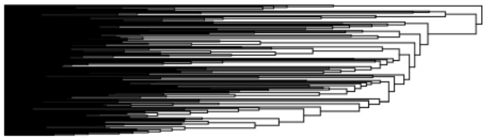

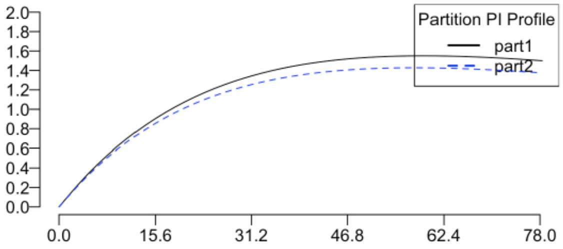

Utility and Suggestions

PI profiles are a powerful heuristic tool, however, there are several properties of this method that deserve consideration by users. Higher values to PI do not mean that a locus has a guarantee of higher bootstrap values or posterior probabilities for a given phylogenetic problem. It simply means that there are more sites predicted to contain phylogenetic information in a given epoch

Moreover, the shape of PI profiles should be carefully considered. Declines in PI profile indicate a rise in homoplasious sites. Townsend and Leuenberger[4] referred to this portion of a PI profile as a 'rainshadow of noise'

Dornburg et al.[5] demonstrated that removing loci whose PI profiles exhibit a sharp decline prior to the root of a tree can have a marked effect on divergence time estimates. For example, in the case of cichlids (below), the estimated times were nearly cut in half

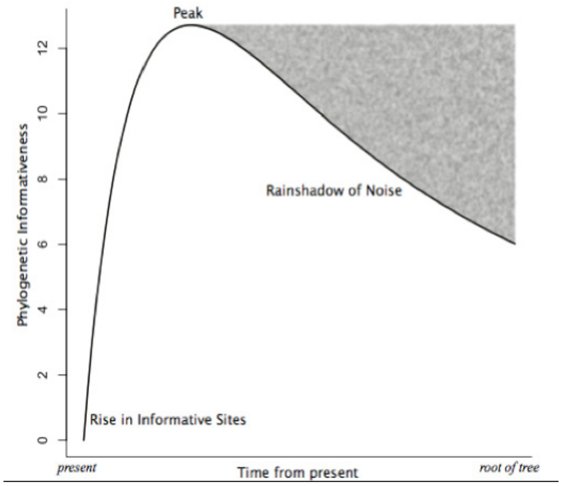

A figure showing the rainshadow of noise, modified from Townsend and Leuenberger[4]

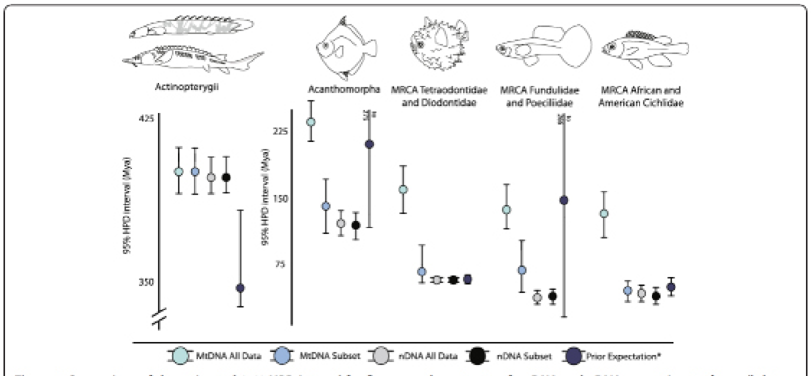

Figure 1 Comparison of the estimated 95% HPD interval for five example contrasts of mtDNA and nDNA age estimates from all data, those from datasets pruned of the saturated partitions, and the prior age expectation based on the effective prior (*analysis run without sequence data): Acanthomorpha (spiny-rayed fishes); MRCA Fundulidae and Poeciliidae (topminnows and livebearers); MRCA African and American cichlids, MRCA Tetraodontidae and Diodontidae (smooth and spiny puffers), and Actinopterygii (ray-finned fishes). Lines indicate bounds of the 95% HPD interval, circles correspond to mean age estimates.

PI profiles are great for predicting trends in a dataset, or for mitigating against homoplasy driven errors in a divergence time studies. However, for predicting topological error a more detailed approach that accounts for time, rate, *and* internode length is required, bringing us to the next section

Section 02

References:

1. Townsend JP. Profiling phylogenetic informativeness. Syst. Biol. 2007;56:222–31.

2. López-Giráldez F, Townsend JP. PhyDesign: an online application for profiling phylogenetic informativeness. BMC Evol. Biol. 2011;11:152.

3. Prum RO, Berv JS, Dornburg A, Field DJ, Townsend JP, Lemmon EM, et al. A comprehensive phylogeny of birds (Aves) using targeted next-generation DNA sequencing. Nature. 2015;526:569–73.

4.Townsend JP, Leuenberger C. Taxon sampling and the optimal rates of evolution for phylogenetic inference. Syst. Biol. 2011;60:358–65.

5. Dornburg A, Alex D, Townsend JP, Matt F, Near TJ. Phylogenetic informativeness reconciles ray-finned fish molecular divergence times. BMC Evol. Biol. [Internet]. 2014;14. Available from: http://dx.doi.org/10.1186/s12862-014-0169-0

.

Section 02

Section

03

Resolution probability quantification

phyinformR

Resolution Probability Quantification

Townsend et al.[1] introduced theory that takes into the account the interplay of site rates, time, and internode length between species divergences to assess the predicted probabilty of data contributing to accurate topological resolution

Use of this method requires three inputs:
site rates, state space, and internode lengths

Site rates are covered at the beginning of section 2. Now we'll introduce the internode lengths and state spaces below

For this walkthrough, we will continue using the avian tree and site rates from Prum et al.[2] that are distributed with PhyInformR. In case these are not in memory already use

```
library(ape)
read.tree(system.file("extdata","Prumetal_timetree.phy",
package="PhyInformR"))->tree
as.matrix(prumetalrates)->rr
```

GETTING STARTED

There are three quantities that phyloInformeR calculates with regard to a specified internode given a set of site rates: Quartet Internode Resolution Probability (QIRP, "Quirp"), Quartet Internode Homoplasy Probability (QIHP, "Quip"), and Quartet Internode Polytomy Probability (QIPP, "Quippy"). Townsend et al.[1] introduced two ways to calculate these quantities: An analytical approximation and a Monte Carlo based solution. Both approaches depend on site rates and two user defined internode lengths, *T* (time from present) and *t* (internode)

Internode Lengths

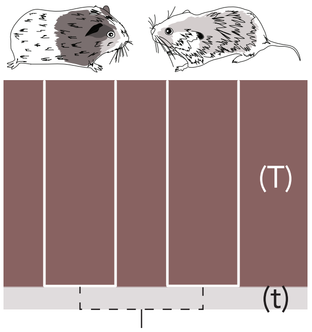

The theory of Townsend et al.[1] defines *T* and *t* based on a phylogenetic quartet with even branch lengths. Under this assumption, *T* is the time from the present to the ancestor of a taxon (red in the example above) and *t*, the focal internode length (grey in the example above). Later in this section we will discuss how to perform similar analyses allowing for uneven quartets

Lets start by calculating approximate solutions for QIRP, QIHP, and QIPP

Using the previous illustration, you should have an idea of what *T* (time) and *t* (internode distance) you will want to use for your data.

For state space, a binary morphological matrix could be assessed by setting the state space to 2 or amino acid (20 or ~5)[1], or other types of data with differing numbers of characters. If you are using nucleotides, despite having a four character states, Simmons et al.[3] have demonstrated the state space to be better modelled using 3 states, so we will go with that for the remainder of this guide.

Here is the implementation using *T*= 100 million year (Ma) and *t*= 0.5 Ma

```
Approximator(100,0.5, rr, 3)
```

```
 Probabilty Correct  Probability Polytomy Probability Incorrect
         0.52747310            0.01256845            0.45995845
```

Alternatively you can use the Monte Carlo simulation approach[1]. Since the simulation is time consuming dependent on the number of simulations and the number of sites, we recommend that you do this on a cluster or while you are doing something else. The output is automatically recorded to file. The input looks similar to the approximation, though now you specify a file name for the probabilities and an image file name. Note that the two files must have different names!

You can also toggle the screen output on and off by setting image to either "TRUE" or "FALSE". The simulation can be run in parallel so

Remember to set your cores appropriately using the registerDoParallel(cores=8)

Here is the function using the same *T* and *t* and 5000 simulations on a subset of sites to save time

```
as.matrix(prumetalrates[1:20000])->rr2
parallel.cluster.signal.noise(100,0.5,rr2,5000,3,filename="test",imagename="testimage",image="FALSE")
```

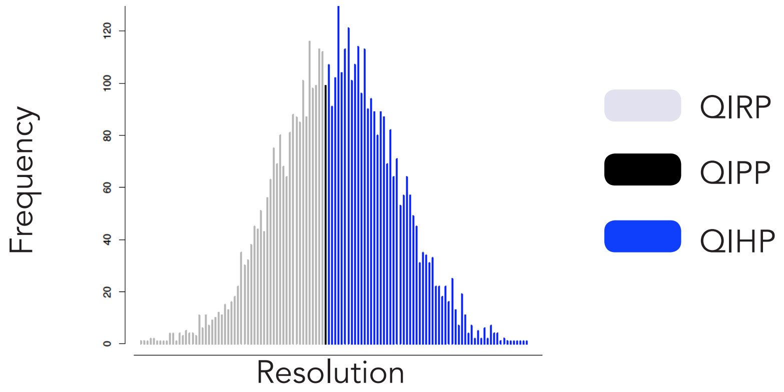

Section 03

Advanced Resolution Probability Quantification

Calculations based on the equations of Townsend et al.[1] make several assumptions

1) equal base frequency distributions in the alignment

2) equal branch lengths within the phylogenetic quartet

3) a Jukes-Cantor model of sequence evolution

Su et al.[3] provided extensions to the equations of Townsend et al.[1] that enable any nucleotide substitution model or distribution of base frequencies and Su and Townsend[4] provided the theoretical framework for relaxing the assumption of even branch lengths

To use these extension, first specify your model settings based on a model selection program such as Modeltest[5], or Partitionfinder[6,7]. Model settings are defined as follows and require changing for different nucleotide substitution models, base frequencies, and branch lengths

For example:

```
a=1
b=1
c=1
d=1
e=1
f=1
Pi_T=0.25
Pi_C=0.25
Pi_A=0.25
Pi_G=0.25
internode<-c(62.4937, 62.4937, 62.4937, 62.4937, 8.9939)
```

Pi_T through Pi_G are the empirical base frequency parameters

IMPORTANT | these must sum up to one | Pi_T + Pi_C + Pi_A + Pi_G = 1

a through f are the relative rate parameters which are defined as follows

$$rCT = rTC = a;\ rAT = rTA = b;\ rTG = rGT = c;\ rCA = rAC = d;\ rCG = rGC = e;\ rAG = rGA = f$$

Section 03

Su et al. [3] provide a reference table for model settings

Number of substitution types	Models of equal equilibrium base frequencies	Models of unequal equilibrium base frequencies
	$\pi_T = \pi_C = \pi_A = \pi_G = \frac{1}{4}$	$\pi_T \neq \pi_C \neq \pi_A \neq \pi_G$
1	JC	F81
	$a = f = b = e = c = d$; equal substitution rate	
2	K2P	HKY
	$a = f \neq b = e = c = d$; transition and transversion	
3	TrNef	TrN
	$a \neq f \neq b = e = c = d$; 2 transitions and transversion	
3	K3P	K3Puf
	$a = f \neq b = e \neq c = d$; transition and 2 transversions	
4	TIMef	TIM
	$a \neq f \neq b = e \neq c = d$; 2 transitions and 2 transversions	
5	TVMef	TVM
	$a = f \neq b \neq e \neq c \neq d$; transition and 4 transversions	
6	SYM	GTR
	$a \neq f \neq b \neq e \neq c \neq d$; 6 unequal substitutions	

internode in the above code is as an object containing the numerical values of branch lengths of T_1, T_2, T_3, T_4, and the internode length t_0 for the four - taxon tree in question

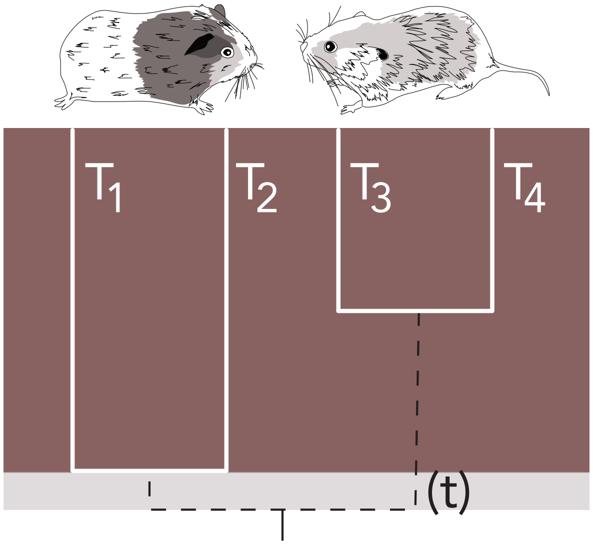

IMPORTANT -T_1 and T_2 must belong to the same sister clade and T_3 and T_4 must belong to the other clade in the *hypothesized* topology of the four - taxon problem in question (see above)

Section 03

Quartet trees do not need to be rooted. All four subtending branches may vary

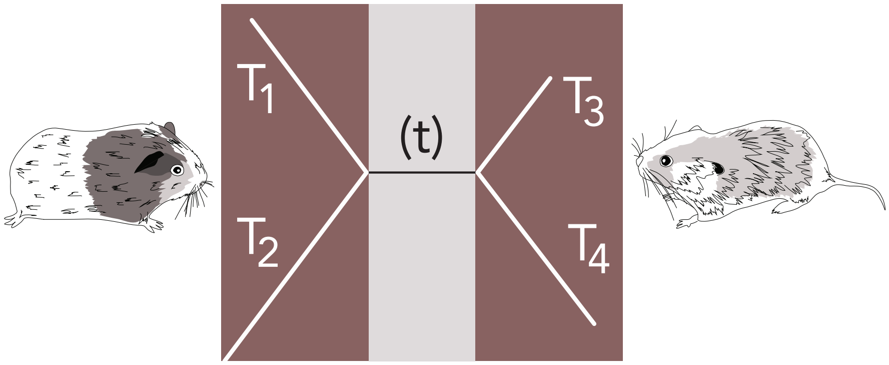

You can quantify QIHP, QIPP, and QIRP with

```
allmodel.signal.noise (a,b,c,d,e,f,internode,Pi_T,Pi_C,Pi_A,Pi_G, rr)
```

From left to right, the three values represent QIHP, QIPP, and QIRP

Posterior Distributions

Since branch length are rarely known with certainty, phyinformR can also be used to calculate QIRP, QIPP, and QIHP values across a distribution of trees such as those obtained from Bayesian analyses.

For this example, we will read in use the sample distribution of bichir trees from a study by Near et al.[8] that is provided with the release

```
library(ape)
read.tree(system.file("extdata","polypterus_trees.phy",
package="PhyInformR"))->tree
as.matrix(rag1)->rate_vector
```

First you will need to specify the quartet of interest. In the bichir dataset, we will look at the clade containing *Polypterus congicus* as this species was not placed with high support in the tree. We define the quartet as follows

```
quart<- c("Polypterus_congicus","Polypterus_bichir","Polypterus_ansorgii" ,"Polypterus_endlicheri" )
```

The remaining objects should be familiar, please review the above and preceeding page if the variable names seem enigmatic. To compute over a distribution of trees, run the function

```
su.bayes(a,b,c,d,e,f,Pi_T,Pi_C,Pi_A,Pi_G,rate_vector,quart,tree)->final
```

This function returns a matrix of internodes and *T* values from the trees and their associated QIHP,QIPP, and QIRP values

Section 03

su.bayes runs in parallel
Remember to set your cores appropriately using registerDoParallel(cores=8)

su.bayes returns a matrix of internodes and *T* values from the trees and their associated QIHP, QIPP, and QIRP values. This matrix can be summarized using

```
plotPosterior(final, plot="QIPs")   #or
plotPosterior(final,plot="violin")
```

Setting plot="qips" returns a density plot of the quartet internode resolution/polytomy/homoplasy probabilities and the internode lengths, while plot = "violin" returns violin plots of the quartet internode resolution/polytomy/homoplasy probabilities and the internode lengths

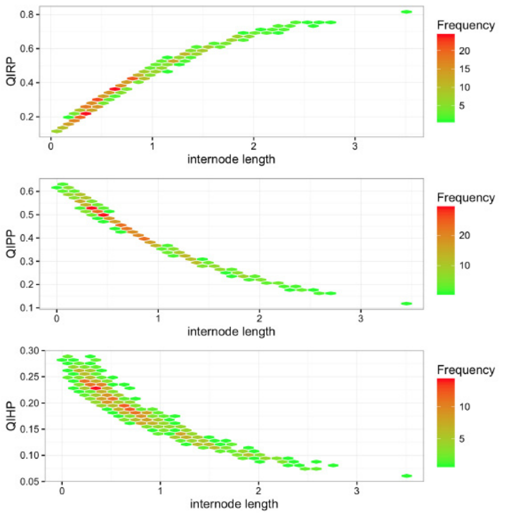

Visualizing the density of calculations reveals a trend that is common in phylogenetic datasets. Both lack of information *and* increased probabilities of convergence misleading inference plague smaller internodes. In this plot we can see that the bulk of the posterior density is in the realm of low QIRP and high QIPP, so we can conclude that the lack of resolution of this clade by this locus is in part predicted to be driven by limited information content

Visualizing the quantiles and kernel density of calculations allows for a additional perspective of how topological and branch length uncertainty influence quantifications. In this case we can see from the box plot of quantiles that QIHP is generally low, but that QIPP is centered near 0.45 with the majority of trees leading to a calculation between about 0.5 and 0.35. The kernel density gives us additional perspective, showcasing somewhat inverted distributions between QIRP and QIPP, with the majority of QIRP values being lower

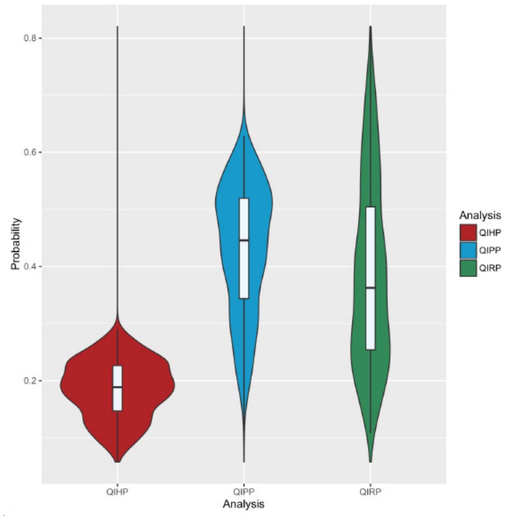

Section 03

Utility and Suggestions

There has long been recognition that loci vary in the amount of information they contain towards resolving specific phylogenetic problems[9]. In particular, loci vary in the degree to which chance convergent or parallel evolution of molecular sites (homoplasy) promote the artificial clustering of lineages in phylogenetic analyses. This variability presents an informatic problem of how to target loci for analyses to efficiently improve phylogenetic accuracy by minimizing homoplasy

Theory by Townsend et al.[1] combined with extensions by Su et al.[3] that generalized the analysis to the General Time Reversible [32, 33] model of nucleotide substitution, and Su and Townsend[4] that relax the assumption of four even subtending branches enable analysis of relative or absolute divergence times in phylogenetic experimental design or for the purpose of data scrutiny[2]

It should be noted that these methods can be used with pilot data on rates from other taxa, including rates computed from deeper-branching phylogenies composed of the closest genome-sequenced organisms to the taxa to be sequenced. Such usage enables markers to be screened for phylogenetic utility prior to sequencing, saving both time and expense. Likewise, using these metrics when designing probe-sets in bioinformatic pipelines using both hypothesized rates and distributions of potential branch lengths should save sequencing costs and result in a more targeted dataset

These methods are also of utility for disentangling sources of incongruence. HGT, lineage sorting, lack of power, or homoplasy can result in lack of strongly supported resolution. At worst, factors such as these can yield strong, but erroneous, support for focal nodes

Assessing if a dataset has high QIPP such as the example in bichirs can illuminate lack of power. Testing if incongruence is driven by lack of power can be particularly informative in a Bayesian framework, providing guidance when faced with the potential pitfalls of the star-tree paradox[10]

Although high QIRP may not always yield high support values, high QIHP or QIPP values should be taken as indicators that analyses are predicted to be misled. These quantifications provide useful metrics for sorting out sources of incongruence, and should become a standard tool in the phylogenomicist's toolbox

Section 03

References:

1. Townsend, J. P., Z. Su, and Y. I. Tekle. Phylogenetic signal and noise: predicting the power of a data set to resolve phylogeny. Systematic Biology 2012; 61:835-849.

2. Prum RO, Berv JS, Dornburg A, Field DJ, Townsend JP, Lemmon EM, et al. A comprehensive phylogeny of birds (Aves) using targeted next-generation DNA sequencing. Nature. 2015;526:569–73.

3. Su, Z., Z. Wang, F. Lopez-Giraldez, and J. P. Townsend. The impact of incorporating molecular evolutionary model into predictions of phylogenetic signal and noise. Phylogenetics, Phylogenomics, and Systematics 2014; 2:11.

4. Su Z, Townsend JP. Utility of characters evolving at diverse rates of evolution to resolve quartet trees with unequal branch lengths: analytical predictions of long-branch effects. BMC Evol. Biol. 2015;15:86.

5. Lanfear, R., B. Calcott, S. Y. W. Ho, and S. Guindon. PartitionFinder: combined selection of partitioning schemes and substitution models for phylogenetic analyses. Molecular Biology and Evolution 2012; 29:1695-1701.

6. Lanfear, R., B. Calcott, D. Kainer, C. Mayer, and A. Stamatakis. 2014. Selecting optimal partitioning schemes for phylogenomic datasets. BMC Evolutionary Biology 14:82.

7. Posada, D., and K. A. Crandall. Modeltest: testing the model of DNA substitution. Bioinformatics 1998;14:817-818.

8. Near, T. J., A. Dornburg, M. Tokita, D. Suzuki, M. C. Brandley, and M. Friedman. 2014. Boom and bust: ancient and recent diversification in bichirs (Polypteridae: Actinopterygii), a relictual lineage of ray-finned fishes. Evolution 68:1014-1026.

9. Graybeal, A. "The Phylogenetic Utility of Cytochrome B: Lessons from Bufonid Frogs" Molecular phylogenetics and evolution 1993;: 256–269.

10. Yang, Z. Fair-balance paradox, star-tree paradox, and Bayesian phylogenetics. Molecular biology and evolution 2007; 24(8), pp.1639-1655.

.

Section 03

Section

04

Advanced visualizations

phyinformR

Advanced visualizations

The quantitative framework of quartet internode calculations lends itself wonderfully to the development of new ways to visualize information in a given dataset. This section highlights graphics from a few recent publications

GETTING STARTED

Hwang et al.[1] depicted QIRP across an entire tree by plotting the QIRP of a marker for each node simultaneously. This plot can be drawn by providing a tree, rate vector, and state space as in section 3. For this example, we will use a single tree from Near et al.[2] along with site rates from the same study

```
library(ape)
read.tree(system.file("extdata","polypterus_trees.
phy",package="PhyInformR"))->tree
as.matrix(rag1)->rate_vector
tree[[1]]->bichir_tree
```

Now that we have our tree we can

```
PlotTreeSI(bichir_tree,rate_vector,3)
```

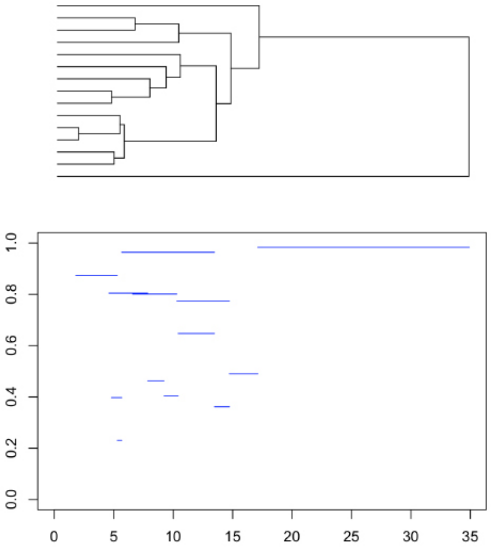

Here the branch lengths (x axis, time) and the blue lines (y axis, QIRP) match up and we can see that a similar trend to the one at the end of the previous section: small recent internode have low QIRP and are predicted to be impacted by homoplasy or contain little information

This approach to visualization can be quite handy when comparing markers

Visualizing Phylogenetic Experimental Design

Say we are interested choosing a new marker to sequence for bichirs.
We can compare the predicted utility of rag1 to candidate markers using this plotting method. In this example we will compare rag1 with the first locus from Prum et al.[3]
We'll start by isolating the locus

```
as.matrix(prumetalrates[1:1594])->candidate.locus
```

Now we can compare to the above plot as follows

```
Plot.Another.TreeSI(bichir_tree, candidate.locus,3,col="red",type=3)
```

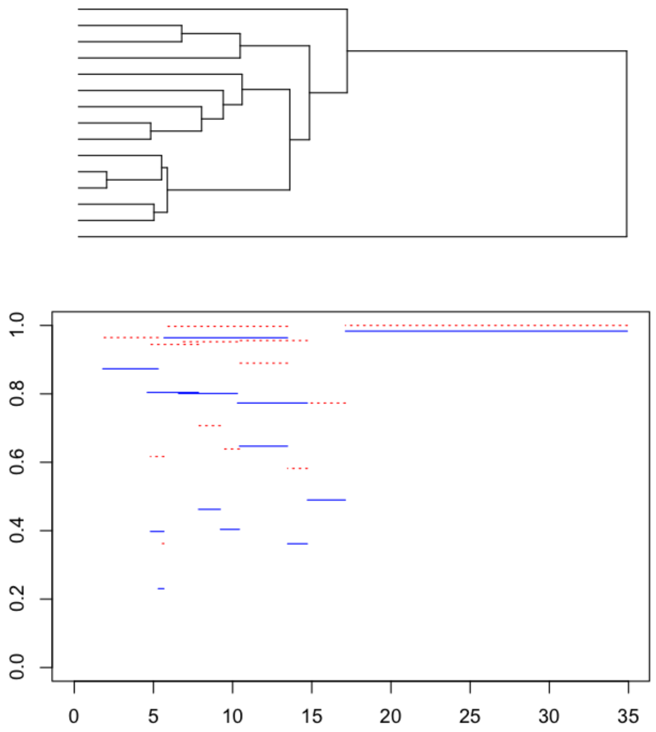

In the above plot QIRP is on the Y axis and time is on the X axis. Blue lines correspond to the QIRP values of the tree internodes for rag1 while red lines correspond to our candidate locus. This visualization reveals that the candidate marker is predicted to be of higher utility for resolving every node in the bichir tree. This sort of visualization can be a great heuristic for choosing probe sets or primers for cost and time effective sequencing of markers. Please note that this function builds on the previous PlotTreeSI function, so leave your graphic window open

This form of visualization also displays overall trends of markers over time, and can help disentangle sources of error in tree inference. However, the above method requires a fixed internode length. Prum et al[3] provided an alternative visualization that accommodates for uncertainty in internode length

Section 04

Predicted Phylogenetic Utility Heatmaps

For this example we will use the files from Prum et al.[3], which are available on Zenodo (DOI 10.5281) https://zenodo.org/record/28343#.WCNweGQrJFQ though you could also substitute site rates from your own markers here

Download the above files and open prumetal_heatmap_rosetta.r that is available in the PhyInformR guthub: https://github.com/carolinafishes/PhyInformR/tree/master/extra_download_files

Navigate to the "Phylogenetic_Informativeness" part of the downloaded Zenodo directory
Change the setwd() function at the top of the rosetta file to the directory path, save, then source

```
setwd("~/yourpath/Zenodo/Phylogenetic_Informativeness")
source("~/yourpathtofile/prumetal_heatmap_rosetta.r")
```

This file translates the rates into locus specific rate matrices for you to explore. For this tutorial we will sort some loci by length to ask whether size of the locus is associated with increased information content. We are going to visualize a random subset of loci for the sake of keeping the tutorial manageable, though feel free to explore the full data further!

```
length(L216)->leL216
length(L182)->leL182
length(L149)->leL149
length(L213)->leL213
length(L184)->leL184
length(L223)->leL223
length(L305)->leL305
length(L8)->leL8
length(L203)->leL203
length(L95)->leL95
length(L41)->leL41
length(L295)->leL295
length(L107)->leL107
length(L163)->leL163
length(L21)->leL21
length(L2)->leL2
length(L325)->leL325
length(L80)->leL80
length(L28)->leL28
c(leL216,leL182,leL149,leL213,leL184,leL223,leL305,leL8,leL203,leL95,
leL41,leL295,leL107,leL163,leL21,leL2,leL325,leL80,leL28)->ll
names(ll)<-c("leL216","leL182","leL149","leL213","leL184","leL223","leL
305","leL8","leL203","leL95","leL41","leL295","leL107","leL163","leL21","
leL2","leL325","leL80","leL28")
sort(ll)
```

Section 04

The guide tree from Prum et al. [3] used relative rates (root height=1), so we are going to look at resolving Neoaves with a crown at .30. We begin by calculating the signal for different internode lengths. Note that the loci were already formatted as matrices in the Prum et al. [3] script.

```
space.maker(allrates,.30,3)->a1
space.maker(L213,.30,3)->a2
space.maker(L182,.30,3)->a3
space.maker(L203,.30,3)->a4
space.maker(L149,.30,3)->a5
space.maker(L2,.30,3)->a6
space.maker(L295,.30,3)->a7
space.maker(L325,.30,3)->a8
space.maker(L107,.30,3)->a9
space.maker(L41,.30,3)->a10
space.maker(L216,.30,3)->a11
space.maker(L28,.30,3)->a12
space.maker(L184,.30,3)->a13
space.maker(L8,.30,3)->a14
space.maker(L95,.30,3)->a15
space.maker(L80,.30,3)->a16
space.maker(L163,.30,3)->a17
space.maker(L21,.30,3)->a18
space.maker(L305,.30,3)->a19
space.maker(L223,.30,3)->a20
```

We now take this output and assemble our heatmaps.

```
rbind(a1,a2,a3,a4,a5,a6,a7,a8,a9,a10,a11,a12,a13,a14,a15,a16,a17,a18,a19,a20)->demo
as.matrix(demo)->demo2
row.names(demo2)<-
c("allrates","L213","leL182","leL203","leL149","leL2","leL295","leL325","leL107","leL41",
"leL216","leL28", "leL184","leL8","leL95","leL80","leL163","leL21","leL305","leL223")
.30/20->by.this
seq(by.this,0.30-0.0001,by=by.this)->lilts
colnames(demo2)<-lilts
heatmap.2(demo2, Colv=F,Rowv=F, scale='none')
```

Section 04

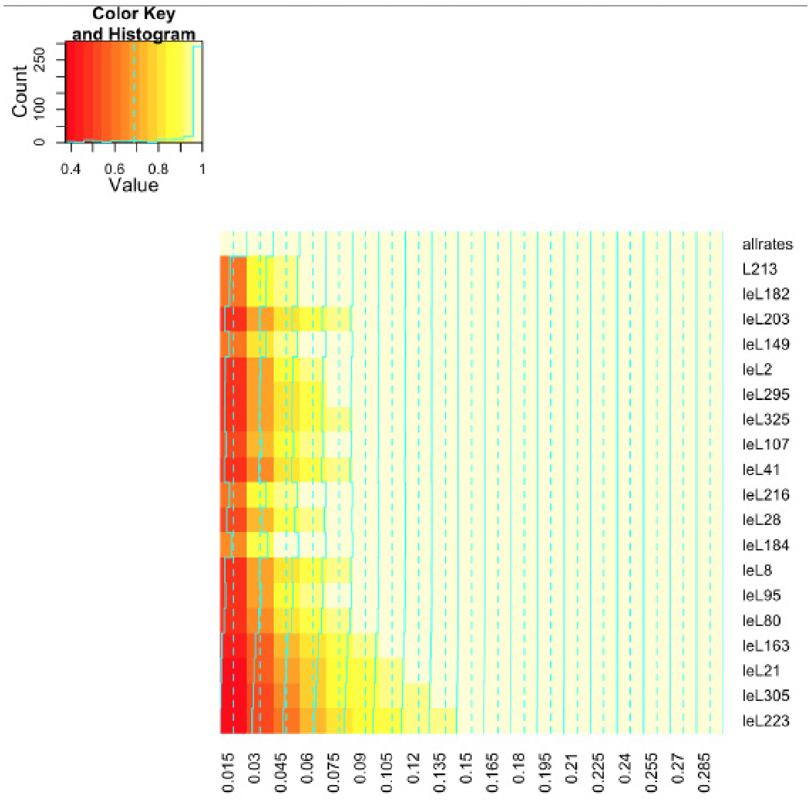

This heatmap shows us the probability of correct resolution for increasingly small internodes. This map can be generated for other loci and the internode distances can be zoomed in on using the space.maker.narrow function

Utility and Suggestions

Phylogenomic scale datasets offer a wealth of information that collectively promise to unlock some of the oldest standing questions in biology. However, the scale of these datasets requires that in addition to the bioinformatics challenges inherent to sorting through data on the order of millions of base pairs, visualizing trends across sorted datasets cannot utilize many conventional visualization methods developed over the last two decades. This problem of scale presents a fundamental challenge to effective scientific discourse and discovery.

The quantitative theory developed to predict quartet internode resolution probabilities offers a means which which we hope will catalyze a new class of phylogenomic visualizations. The equations developed in this framework offer ways to directly quantify a relationship between substitution rate, time, and predicted utility for phylogenetic inference that can applied to any node or nodes across the tree of life and easily catered to individual questions. We hope users fully harness this potential for innovative new visualization and develop new visuals that will enable both scrutiny and discovery of patterns within genome-scale data.

Section 04

References:

1. Hwang J, Jonathan H, Qi Z, Yang ZL, Zheng W, Townsend JP. Solving the ecological puzzle of mycorrhizal associations using data from annotated collections and environmental samples - an example of saddle fungi. Environ. Microbiol. Rep. 2015;7:658–67.

2. Near, T. J., A. Dornburg, M. Tokita, D. Suzuki, M. C. Brandley, and M. Friedman. 2014. Boom and bust: ancient and recent diversification in bichirs (Polypteridae: Actinopterygii), a relictual lineage of ray-finned fishes. Evolution 68:1014-1026.

3. Prum RO, Berv JS, Dornburg A, Field DJ, Townsend JP, Lemmon EM, et al. A comprehensive phylogeny of birds (Aves) using targeted next-generation DNA sequencing. Nature. 2015;526:569–73.

.

Section 04

Acknowledgements

We wish to thank

R. Etter, W.R. Brooks, A. Lamb, U. Ugwuowo, A. Burrus, L. Roupe, L. Lukas, the Townsend, Near, Prum, and Donoghue Lab groups at Yale University and the North Carolina Museum of Natural Sciences Fish Unit for helpful discussions during the development of this software

Two anonymous reviewers for help debugging and enhancing the earlier version of this software.

T. Su for help converting mathematica files into R

Section 05
